# Extraction of Urinary Calculi by a Novel Method

**Published:** 1848-10

**Authors:** 


*Extraction of Urinary Calculi by a Novel Method.*—Dr. Parrish exhibited a specimen of round white calculi, varying in size from a pin's head to a small pea, which had been extracted from the bladder through the eye of a simple flexible catheter of medium size. The whole number withdrawn in this way was about forty, two or three coming away at a time, and generally without pain. As the case furnished some points of unusual interest, Dr. P. stated the history of it, as derived from the patient himself; whose views as to the manner in which these calculi were formed, as well as to the proper method of extracting them, are novel and interesting.

The patient, William Hembel, Esq., President of the Academy of Natural Sciences, is well known to many of the Fellows as a gentleman of varied scientific acquirements, who, although not one of our profession, has devoted much time to medical investigations. He is now past eighty years of age, and has been for some years more or less subject to attacks of painful and difficult urination, from an enlarged prostate gland; this distressing malady has, within the past eighteen months, been considerably aggravated, so that he has been obliged to rely altogether upon the use of the catheter in evacuating the bladder.

In the early part of the present year he suffered to an intense degree, and did not derive the accustomed relief from the use of instruments. The urine was highly ammoniacal, and deposited a large quantity of ropy mucus. In this condition it occurred to him, that a considerable portion of acrid urine, mixed with mucus, must settle down in the bas-fond of the bladder, beyond the reach of the catheter, as ordinarily introduced; and hence that only a portion of its contents was evacuated, the rest remaining behind as a source of renewed irritation. A reference to the anatomical arrangement of the parts confirmed this view, and induced him to resort to a simple expedient to remedy this difficulty. The catheter was introduced by the patient himself in the erect position, and all the fluid contents of the bladder evacuated through it; but, before withdrawing it, the body was bent forward, and the free extremity of the catheter slightly elevated, so as to throw the point of the instrument into the portion of the bladder below the symphysis pubis, at the same time that the palm of the hand was placed between the thighs, in order to allow the fingers to press upon the perineum; gentle motion was now made upon this part, with a view of elevating the depending portion of the bladder, and of thus throwing the mucus which might have settled there, into the eye of the catheter.

By thus striking the perineum, ropy mucus, having an ammoniacal odor, was discharged in considerable quantity, to the great relief of the patient, and fully confirming the correctness of the course adopted. This expedient was successfully resorted to at the close of each operation with the catheter, a portion of mucus always appearing after the urine had been evacuated. It was on one of these occasions that the patient discovered on withdrawing the catheter, that it was blocked up with several small round calculi, which had been thrown into the eye of the instrument, and were thus readily withdrawn without pain. In this way, the whole number of calculi have been at different times extracted; and on only one occasion did any suffering follow; this was occasioned by the calculus being too large to pass within the eye of the instrument, thus presenting a rough surface to the passage, and causing slight laceration of the urethra, with some hemorrhage.

By continuing this plan of emptying the bladder, the irritation of the organ was greatly lessened, the urine improved, and the mucous discharge gradually diminished, leaving the patient in a more comfortable condition than he had experienced for many months. He now believes himself entirely clear from the calculi, and enjoys comparative exemption from suffering, with improved health.

It is evident that the process above described, could not be accomplished with a silver catheter, or with the body in an erect position.

The practical deductions which our venerable friend draws from experience in his own case, may be thus summarily stated:

He believes that much of the suffering attendant upon the disease, (enlarged prostrate) may be caused by the presence of irritating matters, which, from gravity, settle in the concavity of the viscus below the symphysis pubis, and out of the reach of the catheter when introduced in the ordinary way; this mucous secretion, if allowed to remain, forms a vehicle for the collection of solid particles deposited from the urine, and for the formation of calculous concretions. Calculi thus formed, may exist for a long time unsuspected, thus aggravating the spasms of the neck of the bladder, and increasing the incontinence of urine to a great degree. Hence, it is important, in all cases of enlarged prostate, attended with painful urination, thoroughly to evacuate the bladder, either by frequently washing it out, or by the process found so successful in the case now related. To accomplish this, the gum elastic catheter must always be employed, while the position of the body, together with the pressure upon the perineum, in the manner indicated, will be found indispensable to success. The accidental discovery of calculi in the catheter in the present case, seemed to point out the practicability of this method of extracting them, in the early period of their formation, and thus, of preventing a painful and dangerous operation, which might be rendered necessary, if this simple expedient were not adopted.

Should subsequent observation confirm the above views, it must be admitted, that an important addition will have been made to our means of treating one of the most painful diseases to which man is liable, a malady which often renders the latter period of life a season of sorrow and anguish, and causes its victim to look forward to death, as a welcome messenger.—*Ibid.*

